# Supplementary material for: The conformational space of RNase P RNA in solution
Source: Nature. 2024 Dec 18;637(8048):1244–51. doi: 10.1038/s41586-024-08336-6 (PMC11779636; doi:10.1038/s41586-024-08336-6)
Supplement: Supplementary file 1 — Supplementary Figs. 1–8 and Tables 1 and 2. [file 41586_2024_8336_MOESM1_ESM.docx]

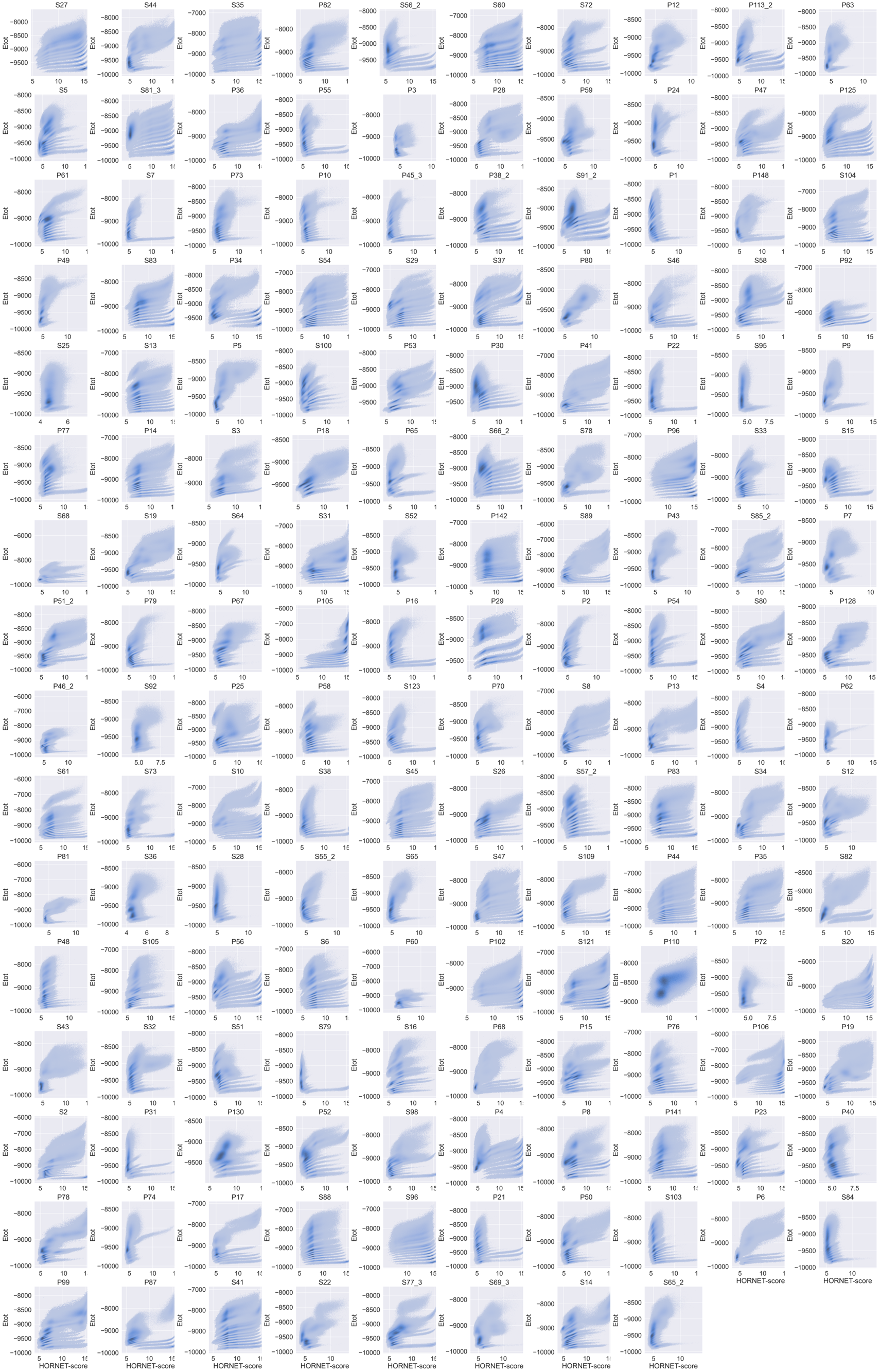


**Supplementary Fig. 1|** Population distribution of the total energy (Etot) as a function of HORNET score (estimated accuracy in terms of RMSD in Å) for the full dynamic fitting trajectory (20 million structures) of each of the 158 individual AFM particles.


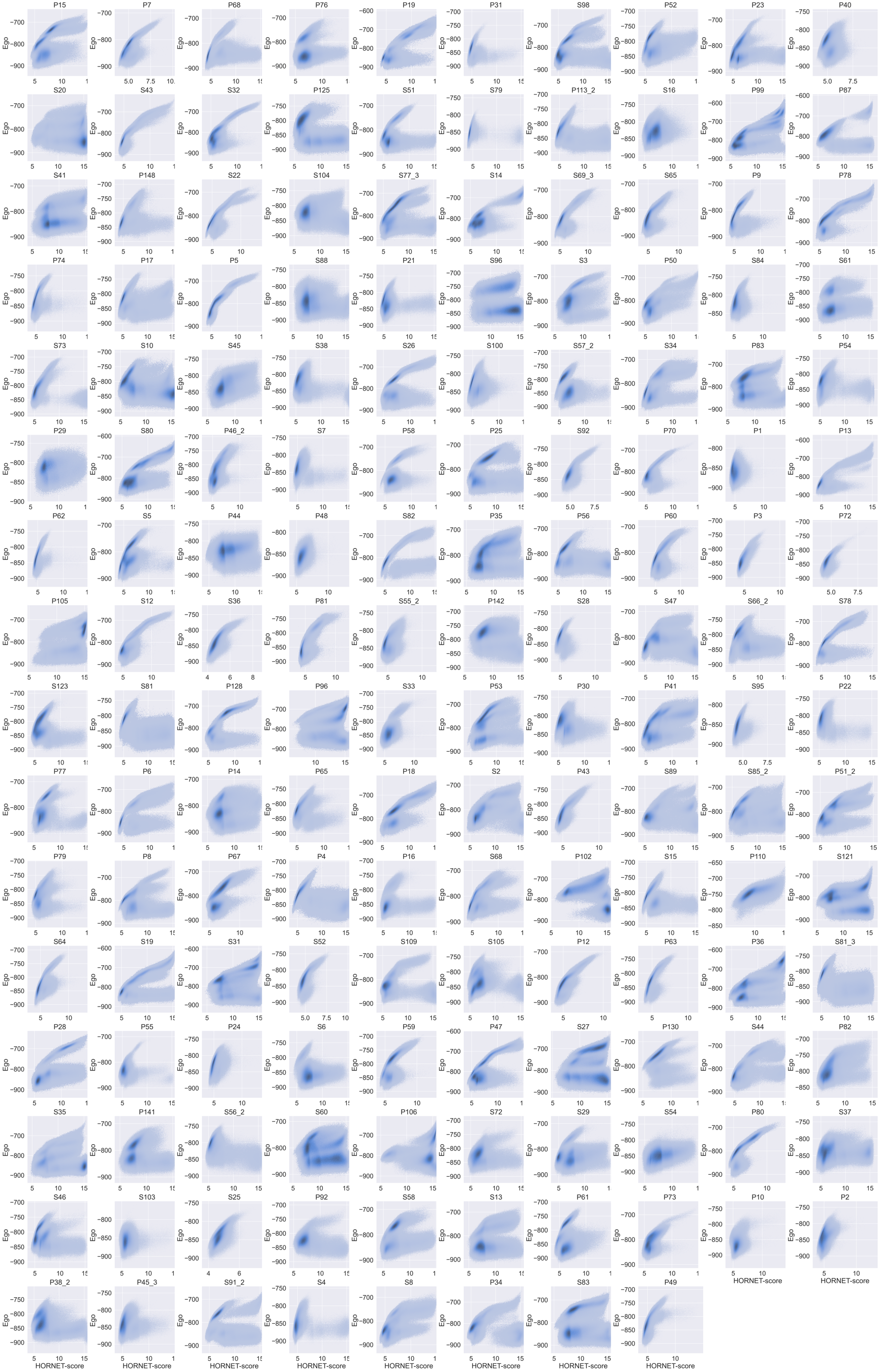


**Supplementary Fig. 2** | Population distribution of the GO energy (Ego) as a function of HORNET score (estimated accuracy in terms of RMSD in Å) for the full dynamic fitting trajectory (20 million structures) of each of the 158 individual AFM particles.


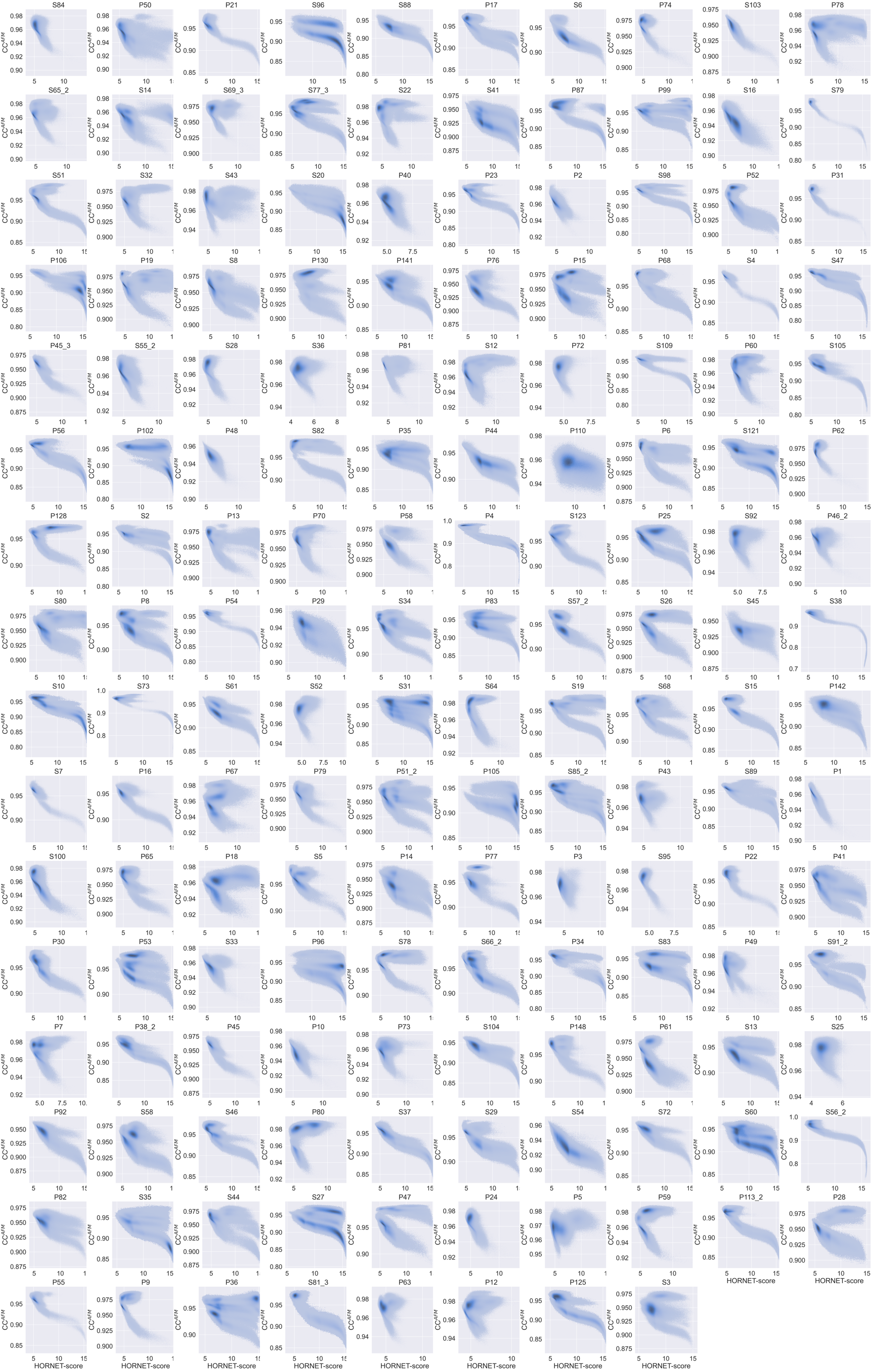


**Supplementary Fig. 3 |** Population distribution of the AFM score (CC^AFM^) as a function of HORNET score (estimated accuracy in terms of RMSD in Å) for the full dynamic fitting trajectory (20 million structures) of each of the 158 individual AFM particles.


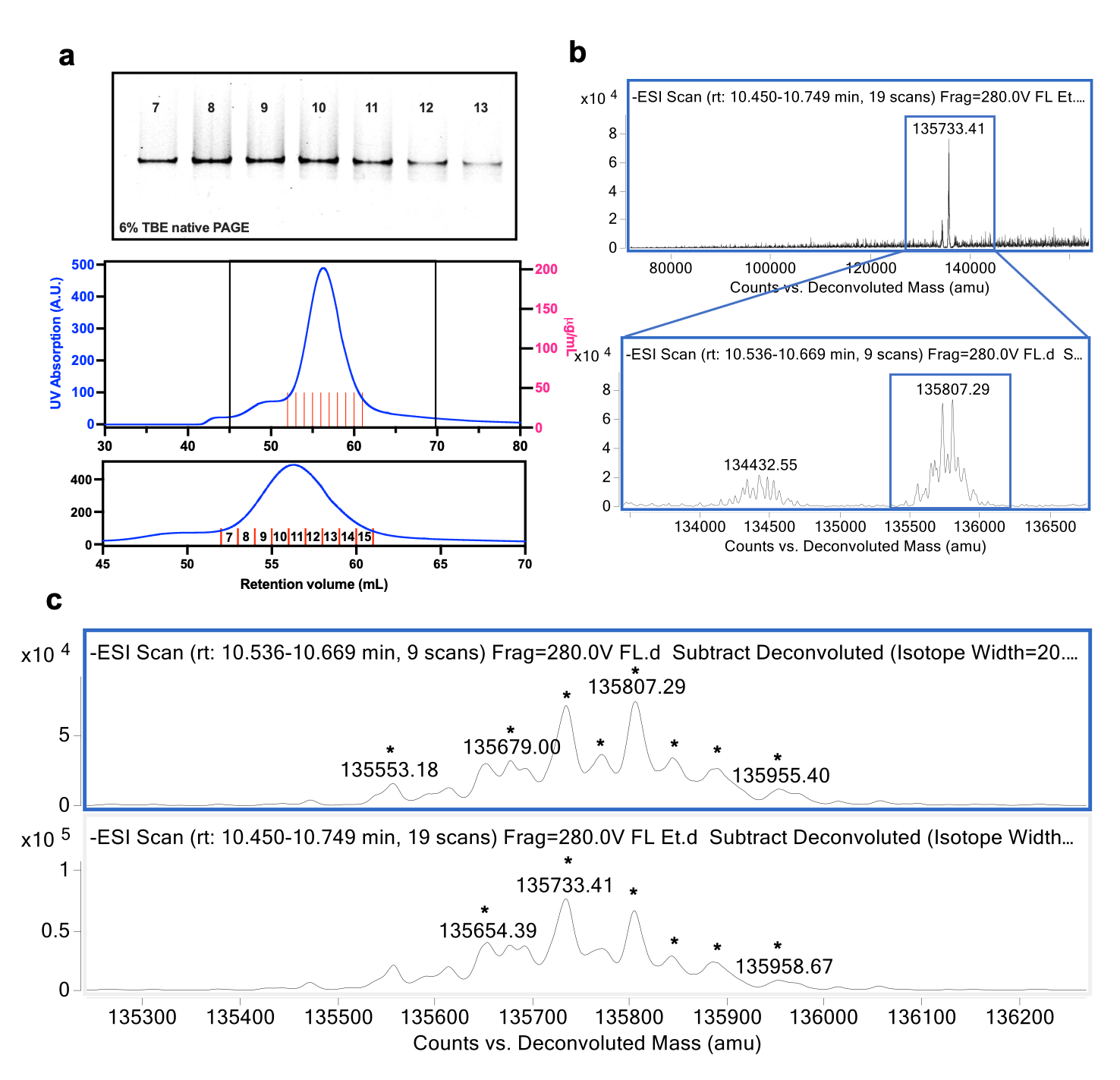


**Supplementary Fig. 4** | RNase P RNA purification by size exclusion chromatography and molecular weight of different ionization states of RNase P RNA confirmed by ESI-MS spectrometry. **a,** Size exclusion chromatogram and 6% native PAGE gel for evaluation of RNA purity and folding. **b**, Deconvoluted ESI-MASS spectrum of RNase P RNA. **c,** Different ionization states of RNase P RNA binding with Mg^2+^.

**
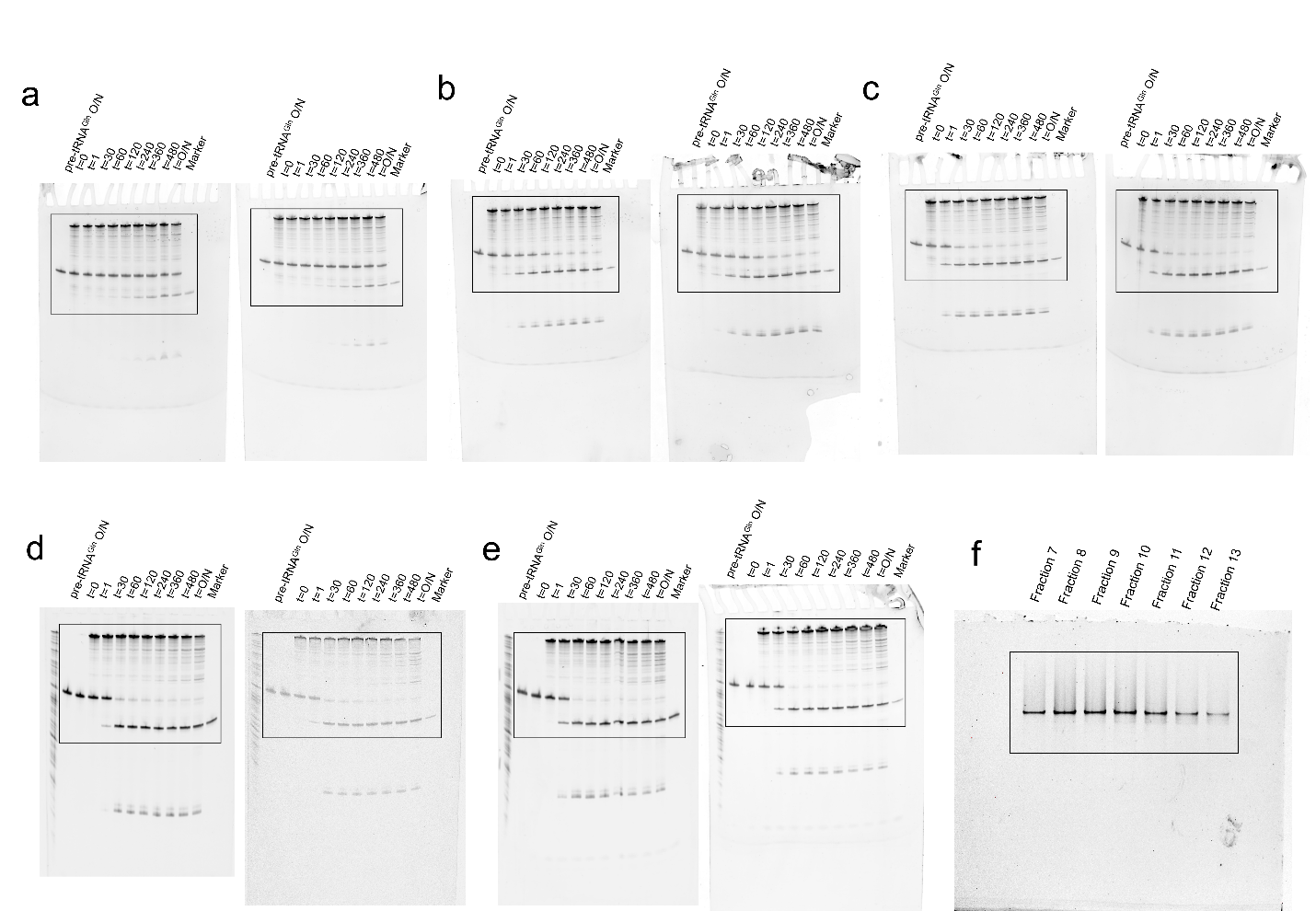
**

**Supplementary Fig. 5| a-e**, Uncropped 8% denaturing PAGE gel duplicates related to RNase P enzymatic assays, conducted in 1.0, 2.5, 5.0, 20, and 50 mM MgCl_2_. The boxed areas are those shown in **Extended Data Fig. 8. f,** Uncropped 6% native TBE PAGE gel of the purified RNase P RNA with the boxed area shown in **Supplementary Fig. 4a**.

**
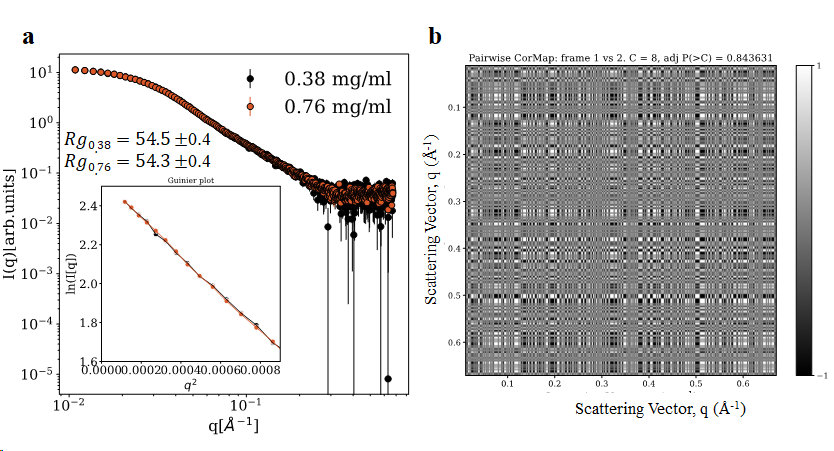
 Supplementary Fig. 6** **|** SAXS data analysis for two different sample concentrations. **a**, SAXS profiles of samples measured at 0.38 mg/ml (black) and 0.76 m/ml (red) scaled by nominal sample concentration, the inset plot shows the respective Guinier plot with determined Rg values. The reduced $\chi^{2}$ test for the profiles is 0.99. **b,** CorMap correlation matrix as a function of q (Å^-1^) with
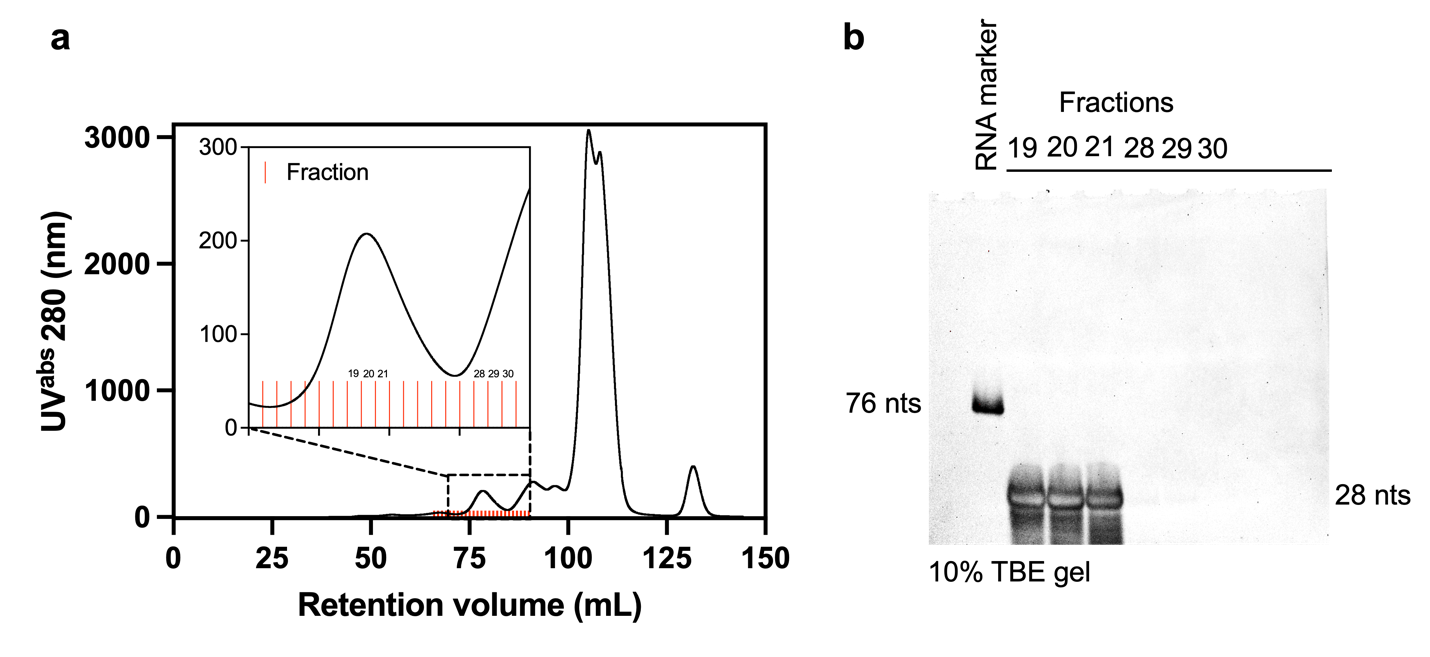
a probability of similarity (P-value) of 0.84.

**Supplementary Fig. 7|** Beet western yellow virus (BWYV) pseudoknot RNA purification. **a,** Size exclusion chromatogram for BWYV pseudoknot RNA purification. Elution fractions used for the isothermal titration calorimetry (ITC) experiment, 19, 20, and 21, are shown in the zoom-in panel. **b**, 10% native PAGE for evaluation of the purity of the BWYV pseudoknot RNA.

**
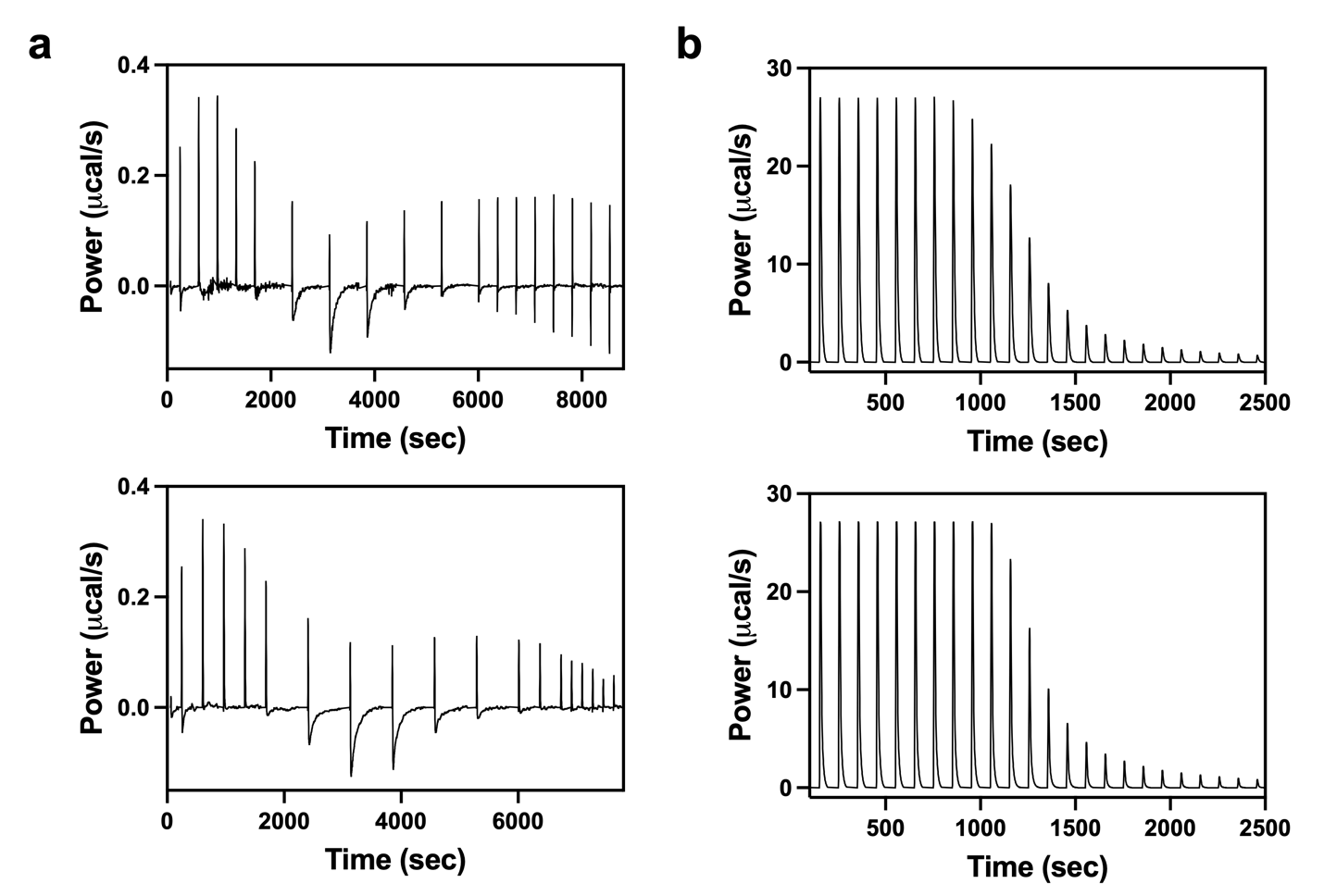
Supplementary Fig. 8 |** Isothermal titration calorimetry (ITC) thermograms. **a,** Duplicates of raw thermograms of Mg^2+^-dependent RNase P RNA compaction. **b,** Duplicates of raw thermograms of Mg^2+^-dependent refolding of BWYV pseudoknot RNA.

**Supplementary Table 1 |** The statistics of the violin plots shown in **Extended Data Fig. 2**

| **Data ID** | **Data size** | **minRMSD** | **maxRMSD** | **meanRMSD** |
| --- | --- | --- | --- | --- |
| P46 | 3564000 | 3.545655 | 14.43933 | 5.679664 |
| P52 | 3564000 | 4.189311 | 15.45458 | 6.580594 |
| P53 | 2277316 | 4.162223 | 15.73201 | 8.005236 |
| P47 | 3564000 | 4.001039 | 15.44401 | 7.193619 |
| P1 | 3564000 | 3.819241 | 14.1605 | 5.052873 |
| P102 | 3564000 | 4.211798 | 15.94399 | 7.911076 |
| P3 | 3564000 | 2.723413 | 11.09227 | 4.763626 |
| P79 | 3443743 | 3.961644 | 15.15345 | 5.844367 |
| P51 | 3564000 | 4.068152 | 15.24603 | 7.309425 |
| P45 | 3564000 | 4.039931 | 15.28959 | 5.634966 |
| P92 | 2574000 | 4.688605 | 15.81023 | 7.676887 |
| P87 | 3564000 | 4.50587 | 16.00973 | 8.043936 |
| P44 | 3564000 | 4.09812 | 16.1217 | 7.958507 |
| P50 | 3564000 | 4.157735 | 15.47663 | 6.438967 |
| P78 | 3564000 | 4.229962 | 15.60152 | 7.900372 |
| P2 | 3564000 | 3.661233 | 12.8462 | 5.069749 |
| P105 | 3564000 | 3.915359 | 15.35686 | 5.276566 |
| P6 | 3564000 | 3.775212 | 15.23921 | 6.343712 |
| P54 | 3564000 | 4.047452 | 16.11578 | 6.000739 |
| P40 | 3564000 | 3.81361 | 9.650901 | 5.139062 |
| P68 | 3564000 | 3.732556 | 15.67548 | 6.036145 |
| P83 | 2970000 | 4.896187 | 15.80649 | 10.91073 |
| P82 | 4752000 | 4.578261 | 15.43518 | 7.318774 |
| P96 | 2376000 | 4.41648 | 15.17875 | 8.501144 |
| P41 | 3564000 | 4.057153 | 15.37154 | 7.652589 |
| P55 | 3564000 | 4.006486 | 15.95193 | 5.678775 |
| P7 | 3564000 | 3.747874 | 10.43798 | 5.016393 |
| P110 | 1386000 | 5.651039 | 15.33592 | 10.01007 |
| P106 | 1980000 | 4.568818 | 15.339 | 8.277196 |
| P5 | 3564000 | 3.642366 | 15.02628 | 6.027244 |
| P43 | 3564000 | 3.729947 | 11.94175 | 5.104299 |
| P80 | 3564000 | 4.181781 | 13.08804 | 6.490073 |
| P81 | 3564000 | 2.791884 | 12.59413 | 5.256427 |
| P56 | 3564000 | 4.10617 | 16.03132 | 8.07054 |
| P42 | 3514208 | 4.557248 | 15.29092 | 7.48813 |
| P4 | 3564000 | 3.845503 | 16.11938 | 8.826895 |
| P113 | 3564000 | 3.815427 | 15.99723 | 6.740056 |
| P148 | 3564000 | 3.858321 | 15.44622 | 5.618737 |
| P19 | 3564000 | 3.614257 | 15.35682 | 7.606038 |
| P25 | 3564000 | 4.287555 | 15.83728 | 7.866812 |
| P31 | 3564000 | 3.897745 | 15.90785 | 5.177479 |
| P30 | 4356000 | 4.094592 | 15.41146 | 6.247449 |
| P24 | 3454555 | 3.353582 | 13.92261 | 5.15888 |
| P18 | 2376000 | 4.410726 | 15.40274 | 8.243431 |
| P32 | 3564000 | 4.114581 | 15.29704 | 6.775122 |
| P33 | 3564000 | 4.251347 | 16.03652 | 8.19848 |
| P23 | 3564000 | 3.991894 | 16.07028 | 6.537827 |
| P22 | 3564000 | 4.298789 | 15.54968 | 5.801148 |
| P36 | 2376000 | 4.352925 | 15.58639 | 7.780581 |
| P34 | 3564000 | 4.067657 | 16.10923 | 8.046536 |
| P35 | 3168000 | 4.704891 | 15.69202 | 8.895588 |
| P21 | 3564000 | 4.070214 | 15.84105 | 5.762487 |
| P141 | 2970000 | 4.64332 | 15.76175 | 7.695265 |
| P38 | 3564000 | 4.226297 | 16.04976 | 7.346815 |
| P10 | 5148000 | 4.07838 | 15.02672 | 6.535778 |
| P13 | 3564000 | 3.792827 | 15.47434 | 5.963379 |
| P12 | 3564000 | 3.819665 | 11.21797 | 5.275494 |
| P16 | 3564000 | 4.238191 | 15.55127 | 6.226111 |
| P17 | 3564000 | 3.930149 | 15.86572 | 6.85445 |
| P15 | 3564000 | 4.201543 | 15.37597 | 7.134492 |
| P29 | 686000 | 4.939807 | 15.40364 | 8.754581 |
| P28 | 3564000 | 4.215995 | 15.19518 | 7.967975 |
| P14 | 3553325 | 4.38692 | 15.97953 | 9.434766 |
| P9 | 3564000 | 3.869122 | 14.65395 | 5.510117 |
| P67 | 3564000 | 4.216508 | 14.00318 | 6.599483 |
| P73 | 3564000 | 4.391793 | 14.86378 | 6.296421 |
| P99 | 3564000 | 3.985938 | 14.54236 | 6.092117 |
| P72 | 3564000 | 3.538936 | 9.068867 | 4.647205 |
| P66 | 3564000 | 3.944696 | 15.45097 | 6.196515 |
| P8 | 3564000 | 4.192075 | 15.728 | 7.577818 |
| P58 | 3564000 | 3.896209 | 15.03701 | 6.628275 |
| P70 | 3564000 | 4.007322 | 14.80785 | 5.879735 |
| P65 | 3564000 | 4.065433 | 15.09422 | 5.756488 |
| P59 | 3564000 | 4.090771 | 13.37035 | 5.977331 |
| P130 | 4158000 | 4.651271 | 15.08103 | 7.756189 |
| P61 | 3564000 | 3.901028 | 14.95023 | 6.29112 |
| P49 | 3564000 | 3.833873 | 14.46487 | 5.181576 |
| P48 | 1764000 | 4.080717 | 15.1881 | 5.907988 |
| P60 | 3564000 | 2.068465 | 13.92113 | 5.920531 |
| P74 | 3564000 | 3.855415 | 14.82018 | 5.081867 |
| P125 | 3564000 | 4.056987 | 15.58791 | 6.873027 |
| P62 | 3564000 | 3.501205 | 14.73134 | 4.862453 |
| P76 | 3564000 | 4.342671 | 15.46252 | 7.027244 |
| P77 | 3564000 | 4.284445 | 15.61087 | 6.80895 |
| P63 | 3564000 | 3.455949 | 11.70712 | 4.904287 |
| S25 | 3564000 | 3.750146 | 7.600447 | 4.713561 |
| S31 | 3564000 | 5.797063 | 16.10135 | 10.84497 |
| S19 | 3564000 | 4.188205 | 15.76979 | 8.420617 |
| S33 | 3564000 | 4.063795 | 14.12085 | 5.82982 |
| S32 | 3564000 | 3.791801 | 14.8842 | 6.26273 |
| S26 | 3564000 | 4.200233 | 15.3793 | 7.25917 |
| S22 | 3564000 | 3.706195 | 14.01586 | 5.600369 |
| S36 | 3564000 | 3.756778 | 8.980188 | 4.763097 |
| S37 | 3564000 | 3.903064 | 15.96228 | 7.591354 |
| S35 | 2178000 | 4.626672 | 15.87878 | 9.186779 |
| S8 | 3564000 | 3.865858 | 15.35475 | 6.646554 |
| S20 | 1264000 | 4.407612 | 15.54605 | 7.525171 |
| S34 | 3564000 | 3.739949 | 15.47371 | 6.587543 |
| S53 | 4338423 | 5.247795 | 15.99259 | 10.96551 |
| S47 | 3564000 | 4.058831 | 16.09917 | 8.371719 |
| S84 | 3256670 | 3.988256 | 14.26569 | 5.488832 |
| S85 | 3564000 | 4.204681 | 16.01711 | 8.337094 |
| S91 | 3564000 | 4.067834 | 15.76853 | 7.844571 |
| S46 | 3564000 | 4.088221 | 15.86166 | 6.587918 |
| S52 | 3564000 | 3.409001 | 9.931259 | 4.917651 |
| S78 | 3381142 | 3.831273 | 15.49815 | 6.733101 |
| S44 | 3564000 | 3.70412 | 15.54968 | 6.638539 |
| S92 | 3036491 | 3.308901 | 9.371401 | 4.978563 |
| S51 | 3564000 | 4.109441 | 15.75464 | 6.435672 |
| S45 | 3564000 | 4.414471 | 16.09539 | 7.007308 |
| S79 | 3317665 | 3.663327 | 16.0831 | 4.844548 |
| S123 | 3564000 | 4.125444 | 15.56035 | 6.160642 |
| S41 | 3564000 | 4.102826 | 15.96452 | 7.176858 |
| S55 | 3564000 | 3.741689 | 12.3873 | 5.090611 |
| S69 | 3564000 | 4.168985 | 14.78766 | 6.615174 |
| S82 | 3492111 | 3.843009 | 15.65724 | 6.612426 |
| S96 | 3564000 | 4.604107 | 15.30625 | 7.681597 |
| S83 | 3564000 | 4.116529 | 14.65322 | 5.47015 |
| S68 | 3564000 | 3.886088 | 15.29682 | 6.311823 |
| S54 | 3564000 | 4.313925 | 15.14083 | 8.023627 |
| S56 | 3564000 | 4.235969 | 16.11008 | 6.657493 |
| S95 | 2959481 | 3.501731 | 9.547112 | 4.497996 |
| S81 | 2503135 | 4.794378 | 15.59546 | 8.300163 |
| S80 | 3445926 | 4.098678 | 15.5966 | 8.096825 |
| S43 | 3564000 | 3.748223 | 14.97443 | 5.789585 |
| S57 | 3564000 | 3.888855 | 15.58077 | 6.621151 |
| S109 | 3564000 | 4.298396 | 16.07773 | 7.152198 |
| S104 | 2772000 | 4.282765 | 15.58191 | 7.732435 |
| S72 | 3168000 | 4.362811 | 15.17405 | 7.911548 |
| S66 | 3564000 | 3.801356 | 15.52784 | 7.009684 |
| S98 | 3071913 | 4.050655 | 16.10482 | 6.87168 |
| S73 | 3564000 | 4.127454 | 16.12826 | 6.280437 |
| S105 | 3564000 | 4.284777 | 16.05895 | 7.103977 |
| S65 | 3564000 | 3.805049 | 13.59969 | 5.360926 |
| S64 | 3564000 | 3.169204 | 13.70734 | 5.249909 |
| S58 | 3564000 | 4.208736 | 15.43335 | 7.323961 |
| S60 | 3564000 | 4.019352 | 15.92319 | 6.474186 |
| S61 | 4356000 | 3.655907 | 15.8859 | 6.628249 |
| S103 | 2178000 | 4.542844 | 15.95001 | 8.643553 |
| S77 | 3564000 | 4.022861 | 16.05082 | 8.037059 |
| S88 | 3564000 | 4.076249 | 15.92953 | 7.648411 |
| S89 | 3133082 | 4.211162 | 16.0657 | 7.860142 |
| S100 | 3564000 | 3.80844 | 15.18452 | 6.291814 |
| S5 | 3564000 | 3.742151 | 15.55188 | 5.980642 |
| S4 | 3564000 | 3.851939 | 15.90772 | 5.416485 |
| S10 | 3484934 | 4.389991 | 16.10712 | 10.06348 |
| S38 | 3564000 | 4.193601 | 16.10933 | 6.260543 |
| S12 | 3564000 | 4.034293 | 14.41016 | 6.1391 |
| S6 | 3480429 | 4.054602 | 15.62317 | 7.696343 |
| S7 | 3564000 | 3.878696 | 15.62342 | 5.355043 |
| S13 | 3564000 | 3.989982 | 16.09263 | 7.105692 |
| S3 | 2574000 | 4.8116 | 15.32553 | 8.057585 |
| S2 | 3564000 | 4.289593 | 16.10628 | 8.626066 |
| S16 | 2352000 | 4.282719 | 14.10715 | 6.004381 |
| S14 | 3564000 | 4.206654 | 15.69244 | 7.395865 |
| S28 | 3564000 | 4.068576 | 12.53733 | 5.048791 |
| S29 | 3564000 | 4.034244 | 15.47617 | 7.475823 |
| S15 | 3564000 | 3.90211 | 15.98889 | 6.493836 |

**Supplementary Table 2 |** Structurally invariant core residues.

| Residue Number | Structural Variation (Å^3^) | Sec. Structure position |
| --- | --- | --- |
| 22 | 91.4 | P2 |
| 23 | 72.9 | P2 |
| 24 | 63.2 | P2 |
| 25 | 68.1 | P2 |
| 26 | 97.9 | P2 |
| 43 | 77.8 | P2 |
| 44 | 36.6 | P2 |
| 45 | 23.2 | P2 |
| 46 | 15.7 | P2 |
| 47 | 19.4 | P4 |
| 48 | 17.7 | P4 |
| 49 | 10.9 | P4 |
| 50 | 3.3 | P4 |
| 51 | 2.7 | P4 |
| 52 | 8.4 | P4 |
| 53 | 14.0 | P4 |
| 54 | 4.6 | P4 |
| 55 | 1.5 | P4 |
| 56 | 1.13 | P4 |
| 58 | 0.35 | P4 |
| 59 | 40.5 | P4 |
| 276 | 112.1 | P15.1-P15.2 |
| 277 | 104.9 | P15.1-P15.2 |
| 330 | 129.5 | J2-J15.2 |
| 331 | 59.1 | J2-J15.2 |
| 332 | 33.5 | J2-J15.2 |
| 333 | 51.3 | J2-J15.2 |
| 334 | 137.5 | J2-J15.2 |
| 335 | 146.3 | J2-J15.2 |
| 336 | 55.1 | J2-J15.2 |
| 337 | 27.8 | J2-J15.2 |
| 338 | 25.4 | J2-J15.2 |
| 339 | 43.9 | P2 |
| 340 | 84.2 | P2 |
| 341 | 121.2 | P2 |
| 385 | 47.8 | P4 |
| 386 | 21.4 | P4 |
| 387 | 9.7 | P4 |
| 388 | 6.3 | P4 |
| 389 | 5.3 | P4 |
| 390 | 3.9 | P4 |
| 391 | 0.54 | P4 |
| 397 | 0.83 | P4 |
| 398 | 2.0 | P4 |
| 399 | 7.4 | P4 |
| 400 | 12.6 | P4 |
| 401 | 30.8 | P4 |

**Supplementary Video 1 |** Structural motions derived from PCA (Principal Component 1) using the 158 RNase P conformers.

**Supplementary Video 2 |** Structural motions derived from PCA (Principal Component 2) using the 158 RNase P conformers.

**Supplementary Video 3 |** Structural motions derived from PCA (Principal Component 3) using the 158 RNase P conformers.

**Supplementary Video 4 |** Structural motions derived from PCA (Principal Component 4) using the 158 RNase P conformers.

**Supplementary Video 5 |** Structural motions derived from PCA (Principal Component 5) using the 158 RNase P conformers.

**Supplementary Video 6 |** Conformational transition between concave and flat conformers of RNase P RNA.
