# Supplementary material for: The conformational space of RNase P RNA in solution
Source: Nature. 2024 Dec 18;637(8048):1244–51. doi: 10.1038/s41586-024-08336-6 (PMC11779636; doi:10.1038/s41586-024-08336-6)
Supplement: Supplementary file 2 — Reporting Summary [file 41586_2024_8336_MOESM2_ESM.pdf]

nature portfolio

Corresponding author(s): Yun-Xing Wang

Last updated by author(s): Oct 13, 2024

Reporting Summary

Nature Portfolio wishes to improve the reproducibility of the work that we publish. This form provides structure for consistency and transparency in reporting. For further information on Nature Portfolio policies, see our Editorial Policies and the Editorial Policy Checklist.

Statistics

For all statistical analyses, confirm that the following items are present in the figure legend, table legend, main text, or Methods section.

n/a	Confirmed	
☐	☒	The exact sample size (*n*) for each experimental group/condition, given as a discrete number and unit of measurement
☐	☒	A statement on whether measurements were taken from distinct samples or whether the same sample was measured repeatedly
☒	☐	The statistical test(s) used AND whether they are one- or two-sided<br>*Only common tests should be described solely by name; describe more complex techniques in the Methods section.*
☐	☒	A description of all covariates tested
☐	☒	A description of any assumptions or corrections, such as tests of normality and adjustment for multiple comparisons
☐	☒	A full description of the statistical parameters including central tendency (e.g. means) or other basic estimates (e.g. regression coefficient) AND variation (e.g. standard deviation) or associated estimates of uncertainty (e.g. confidence intervals)
☒	☐	For null hypothesis testing, the test statistic (e.g. *F*, *t*, *r*) with confidence intervals, effect sizes, degrees of freedom and *P* value noted<br>*Give P values as exact values whenever suitable.*
☒	☐	For Bayesian analysis, information on the choice of priors and Markov chain Monte Carlo settings
☒	☐	For hierarchical and complex designs, identification of the appropriate level for tests and full reporting of outcomes
☐	☒	Estimates of effect sizes (e.g. Cohen's *d*, Pearson's *r*), indicating how they were calculated

Our web collection on statistics for biologists contains articles on many of the points above.

Software and code

Policy information about availability of computer code

Data collection	Asylum atomic force microscope was used to acquire AFM images, and CafeMol 3.02 was used for generating the Dynamic Fitting data.<br>The SAXS data were recorded using the in-house instrument at the NCI SAXS core facility (BioSAXS-2000, Rigaku) in the Center for Structural Biology.<br>Dynamic light scattering were collected at NCI Biophysics Resource facility in the Center for Structural Biology using DynaPro Plate Reader III Dynamic Light Scattering instrument (Wyatt Technologies).<br>Data acquisition of ESI-MASS spectrum were performed on 6520 Accurate-Mass Q-TOF LC/MS system equipped with a dual electro-spray source at NCI Biophysics Resource facility in the Center for Structural Biology.

Data analysis

Novel software for data analyses in this study:
HORNET - Holistic RNA Structure Determination (v1.0.0). Zenodo. https://doi.org/10.5281/zenodo.10637777
Script for PCA and SAXS ensemble fitting and chi2 mapping can be found at https://home.ccr.cancer.gov/csb/pnai/data/conformational_space/Conf_space_RNasePRNA/scripts_analysis/.

Other existing softwares used in this study:
Gwyddion 2.65 for AFM data processing.
ATSAS 3.2.1 software package was used for SAXS data analysis.
ImageJ 1.54h was used to quantify the pre-tRNA digested band intensity on PAGE gel.
Mass Hunter Qualitative Analysis software (version B.07.00) was used for ESI-MASS data analysis and deconvolution of mass spectra.
JalView (2.11.2.7) was used for phylogenetic analysis of multiple sequence alignments of RNase P RNA.
PyMol 2.5.4 and UCSF Chimera 1.16 were used to visualize and display RNA structure for figure preparation.
COOT (0.9.8.95.EL) was used for RNA structure regularization.

For manuscripts utilizing custom algorithms or software that are central to the research but not yet described in published literature, software must be made available to editors and reviewers. We strongly encourage code deposition in a community repository (e.g. GitHub). See the Nature Portfolio guidelines for submitting code & software for further information.

Data

Policy information about availability of data

All manuscripts must include a data availability statement. This statement should provide the following information, where applicable:

- Accession codes, unique identifiers, or web links for publicly available datasets
- A description of any restrictions on data availability
- For clinical datasets or third party data, please ensure that the statement adheres to our policy

Data available for public access at: https:/home.ccr.cancer.gov/csb/pnai/data/HorNet/ and
https://home.ccr.cancer.gov/csb/pnai/data/conformational_space/Conf_space_RNasePRNA/scripts_analysis/

Rfam database for RNase P RNA phylogenetic analysis: https://rfam.org/family/RF00011

SAXS data are available at SAS data bank https://www.sasbdb.org/project/2201/b9y8c4b6qf
and the SASBDB accession codes: SASDTA7, SASDTB7, SASDTC7, SASDTD7, SASDTE7, SASDTF7, SASDTG7, SASDTH7, SASDTJ7.

Research involving human participants, their data, or biological material

Policy information about studies with human participants or human data. See also policy information about sex, gender (identity/presentation), and sexual orientation and race, ethnicity and racism.

Reporting on sex and gender	N/A
Reporting on race, ethnicity, or other socially relevant groupings	N/A
Population characteristics	N/A
Recruitment	N/A
Ethics oversight	N/A

Note that full information on the approval of the study protocol must also be provided in the manuscript.

Field-specific reporting

Please select the one below that is the best fit for your research. If you are not sure, read the appropriate sections before making your selection.

☒ Life sciences ☐ Behavioural & social sciences ☐ Ecological, evolutionary & environmental sciences

For a reference copy of the document with all sections, see nature.com/documents/nr-reporting-summary-flat.pdf

Life sciences study design

All studies must disclose on these points even when the disclosure is negative.

Sample size

161 AFM single-molecule images for illustration of RNase P RNA conformational space. following PCA validates the sample size is sufficient to cover all covariances. The resulting 158 recapitulated structures are thus used for SAXS analysis.
SAXS and dynamic light scattering profiles were recorded at 9 different Mg2+ concentrations.
Enzymatic duplicates assays for RNase P RNA were conducted at 5 different Mg2+ concentrations.

114 RNase P RNA sequences with well-defined secondary structure from different bacterial species were used for multiple sequence alignment.

Data exclusions	AFM particle images that were overlapped with others or aggregate were not used for structural determination and 3 particles that are not converge in reasonable computational time were excluded.
Replication	Enzymatic assays for RNase P and SAXS data were duplicated and repeated after 24 hours. The results were successful obtained.
Randomization	Randomized analyses were performed to observe the convergence of SAXS data fitting using the 158-particle ensemble.
Blinding	This approach was not applicable in this study where had no need of performed blind validation since AI model was trained, which is not amenable to blinding.

Reporting for specific materials, systems and methods

We require information from authors about some types of materials, experimental systems and methods used in many studies. Here, indicate whether each material, system or method listed is relevant to your study. If you are not sure if a list item applies to your research, read the appropriate section before selecting a response.

Materials & experimental systems

n/a	Involved in the study
☒	☐ Antibodies
☒	☐ Eukaryotic cell lines
☒	☐ Palaeontology and archaeology
☒	☐ Animals and other organisms
☒	☐ Clinical data
☒	☐ Dual use research of concern
☒	☐ Plants

Methods

n/a	Involved in the study
☒	☐ ChIP-seq
☒	☐ Flow cytometry
☒	☐ MRI-based neuroimaging
